# Altered Viral Replication and Cell Responses by Inserting MicroRNA Recognition Element into PB1 in Pandemic Influenza A Virus (H1N1) 2009

**DOI:** 10.1155/2015/976575

**Published:** 2015-02-19

**Authors:** Xiaoyue Shen, Wenkui Sun, Yi Shi, Zheng Xing, Xin Su

**Affiliations:** ^1^Department of Respiratory Medicine, Jinling Hospital, Nanjing University School of Medicine, Nanjing, Jiangsu 210002, China; ^2^Medical School and the State Key Laboratory of Pharmaceutical Biotechnology, Nanjing University, Nanjing, Jiangsu 210008, China; ^3^Veterinary and Biomedical Sciences, College of Veterinary Medicine, University of Minnesota, Twin Cities, 1971 Commonwealth Avenue, Saint Paul, MN 55108, USA

## Abstract

*Objective*. MicroRNAs (miRNAs) are endogenous noncoding RNAs that spatiotemporally modulate mRNAs in a posttranscriptional manner. Engineering mutant viruses by inserting cell-specific miRNA recognition element (MRE) into viral genome may alter viral infectivity and host responses in vital tissues and organs infected with pandemic influenza A virus (H1N1) 2009 (H1N1pdm). *Methods*. In this study, we employed reverse genetics approach to generate a recombinant H1N1pdm with a cell-specific miRNA target sequence inserted into its PB1 genomic segment to investigate whether miRNAs are able to suppress H1N1pdm replication. We inserted an MRE of microRNA-let-7b (miR-let-7b) into the open reading frame of PB1 to test the feasibility of creating a cell-restricted H1N1pdm virus since let-7b is abundant in human bronchial epithelial cells. *Results*. miR-let-7b is rich in human bronchial epithelial cells (HBE). Incorporation of the miR-let-7b-MRE confers upon the recombinant H1N1pdm virus susceptibility to miR-let-7b targeting, suggesting that the H1N1pdm and influenza A viruses can be engineered to exert the desired replication restrictive effect and decrease infectivity in vital tissues and organs. *Conclusions*. This approach provides an additional layer of biosafety and thus has great potential for the application in the rational development of safer and more effective influenza viral vaccines.

## 1. Introduction

Influenza A viruses are a major group of pathogens causing respiratory tract infections. Seasonal influenza epidemics usually claim up to tens of thousands of lives annually, especially in the elderly and the young in countries such as China and USA. Since its first identification in North America in April 2009, the pandemic influenza A virus (H1N1) 2009 (H1N1pdm) has resulted in hundreds of thousands of confirmed cases worldwide, giving rise to the first pandemic in the 21st century [1–5]. Past infections cannot effectively protect humans from infections with new strains emerging from constant viral genome mutations including antigenic drift and reassortment, which can obtain resistance to antiflu drugs such as Tamiflu [6, 7]. Nevertheless, vaccination is still the recommended approach for prevention of influenza virus infection or reducing severity of illness. The efficacy of influenza vaccines in protection, however, has been in question, which obviously demands the invention of new generations of vaccines, both inactive and attenuated, to better combat influenza epidemics and outbreaks in the future [8, 9].

The distribution of many cellular small noncoding microRNAs (miRNAs) is cell- and tissue-specific [10–12]. When respective miRNA binding targets are loaded into the viral nucleic acid sequence, vRNA or mRNA of the viral genes will be recognized and targeted by the cellular miRNAs and subsequently cleaved or posttranscriptionally repressed [13, 14]. The idea to attenuate influenza A virus by insertion of miRNA target sequence has been already reported by Perez et al. [15]. The study has some deficiencies. The amount of miR-93 in the lung tissue is not high. NP-protein is the influenza virus protein structure, not the key to viral replication proteins. The mutant viruses constructed in the study cannot be suppressed effectively to replicate in the lung tissue. Attenuated influenza virus as a vaccine is generally used by the way of nasal inhalation. In this study, we aimed to develop and use a novel approach for lung-specific attenuated influenza A viruses by exploiting miRNA-mediated gene silencing in the initial link of the synthesis of virus RNA. As shown in Miranda online database (http://www.microrna.org/) and some references, microRNA-let-7b is highly expressed in lung tissues and airway epithelial cells and lowly expressed in HEK293 cells and chicken embryos [16–18]. PB1 is one of the three subunits of the RNA-dependent RNA polymerase. We inserted an miRNA recognition element (MRE) of let-7b into PB1 to create a lung-restricted H1N1pdm virus since let-7b is abundant in bronchial epithelial cells. Because the 3′ noncoding region of the influenza virus RNA is relatively short, in this study, we report our incorporating microRNA response elements (MREs) for miRNA let-7b into the open reading frame of the PB1 gene and generate mutant viruses [15]. We found that the mutant viruses can be suppressed to replicate in bronchial epithelial cells which highly express let-7b, thus producing an attenuated phenotype. The mutant viruses, however, showed no attenuation in HEK293 cells in which let-7b is largely absent. We further show that attenuation is due to the interaction between the cellular miRNA and the viral target. This approach provides a new but general mechanism for controlling viral replication in specific tissues, which will enhance our rational design of stable and attenuated vaccines. The vaccines can elicit effective immunity against viral infections.

## 2. Methods

### 2.1. Viruses and Cells Culture

The influenza A virus, A/Nanjing/NJU-108/2009 (H1N1) isolated in China in 2009, was previously described and thereafter referred to as H1N1pdm [19, 20]. The inoculation and propagation of the influenza A virus were described previously [21].

### 2.2. Plasmids

**Figure 1:**
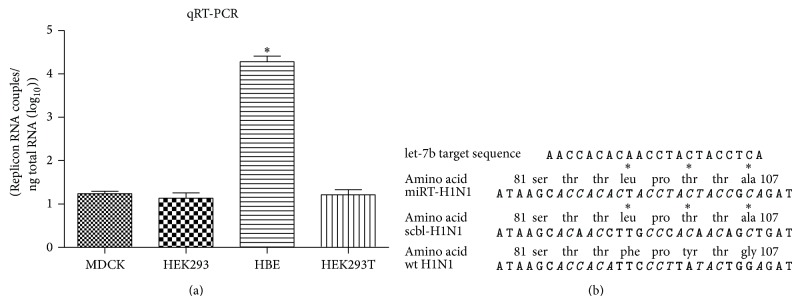
Engineering viruses with restricted cell tropism. (a) Representative quantitative RT-PCR analysis of let-7b miRNA expression levels observed in cell lines (MDCK, HEK293, HEK293T, and HBE). Error bars denote means ± SD from triplicate experiments; similar results were obtained in three independent experiments. ^*^ *P* < 0.05. (b) Schematic of cDNA base substitutions generated to transform miRNA recognition element in scbl-H1N1 or miRT-H1N1. MREs that are complementary to let-7b are in italic type, and mismatched parts are in bold. ∗ denotes amino acid substitutions.

The genomic segments of the H1N1pdm have been cloned into a plasmid, pDP2000, which harbors PB2, PB1, PA, HA, NP, NA, M, and NS cDNA, respectively, for reverse genetics approach to generate recombinant viruses described previously [21–23]. Mutant PB1 cDNA constructs, pDP2000-mu-PB1 and pDP2000-scbl-PB1 plasmids, were generated using the following strategies: the cDNA of A/Nanjing/NJU-108/2009 (H1N1) PB1 gene (GeneBank accession number gi 364023763) was amplified in two parts by polymerase chase reaction (PCR) using primers designed to include miRNA target sequences, specific for let-7b, to be mu-PB1 as indicated in Figure 1(b). To construct mu-PB1 and scbl-PB1, the 5′ and 3′ terminal parts of PB1 cDNA were amplified separately, purified in 1.5% agarose gel, and linked by an overlapping extension PCR method. Subsequently mu-PB1 and scbl-PB1 were subcloned into pDP2000 to produce pDP mu-PB1 and pDP scbl-PB1. The primers' sequences were determined as previously described [24]. The primers' sequences were designed to include the miRNA target sequences as follows: MREs that are complementary to let-7b are in italic type, and mismatched parts are in bold, mu-PB1: forward:* 5*′-ATGCCATAAG**C** *ACCACAC* **T** *ACCTACTACC* **G** *CA*GATCCTCCAT-*3*′; reverse:* 5*′*-*ATGGAGGATC*TG* **C** *GGTAGTAGGT* **A** *GTGTGGT* **G**CTTATGGCAT-3′, scbl-PB1: forward: 5′- ATGCCATAAG**C** *AC* **A** *AC* **CTTG** *CC* **C** *AC* **A** *AC* **AG** *C* **T**GATCCTCCAT-3′; reverse: 5′-ATGGAGGATC**A** *G* **CT** *GT* **T** *GT* **G** *GG* **CAAG** *GT* **T** *GT* **G**CTTATGGCAT-3′.

To construct pmir-FLuc-REPORT series sensors, the part of mu-PB1 which includes the miR-let-7b-MRE was PCR-cloned into XhoI and NotI of pmir-report in 3′ UTR (Ribobio, Guangzhou, China), together with the insertion of an scbl-PB1 or wt-PB1 PCR-fragment derived from either pDP mu-PB1, pDP scbl-PB1, or pDP wt-PB1, resulting in pmir-mu-PB1, pmir-scbl-PB1, and pmir-wt-PB1, respectively (Figure 7(a)). The sequences of the primers used in this study are listed as follows: forward: 5′-CCGCTCGAGATTCTAACTAAGGCAAACCATTTGAAT-3′; reverse: 5′-GAATGCGGCCGCCCCTTTTGTCCAAGATTCAGTAT-3′.

### 2.3. Transfection, Titration, and Sequence Analysis of Recombinant Viruses

Reassortant virus were generated by cotransfecting mixed HEK293T and MDCK cultures with the eight plasmids using Lipofectamine 2000 (Invitrogen) to make miRT-H1N1 and scbl-H1N1, containing miRT-PB1 and scbl-PB1, respectively, following the procedure described previously [21]. The titration of recombinant viruses was also described [21]. To verify the presence of the inserted miRNA target sequences in the virus genome, viral RNA was prepared from the allantoic fluid, reverse-transcribed to cDNA prior to synthesis of the gene segments by PCR using a PrimeSTAR max DNA polymerase kit (Takara, Tokyo, Japan) with segment-specific primers [24]. Sequencing analyses were performed to confirm the alternation of the genomic sequences in either miRT-H1N1 or scbl-H1N1 as designed with an Illumina HiSeq 2000 platform at the Nanjing Genscript Corporation (Nanjing, China; http://www.genscript.com.cn/).

### 2.4. miRNA Mimics

miRNA mimics were purchased from GenePharma, Shanghai, China. An miRNA mimic corresponding to* Caenorhabditis elegans* miRNA with no predicted miRNA target elements (miRTs) in mammalian cells was used as control according to the manufacturer. miRNA inhibitor (5′AACCACACAACCUACUACCUCA 3′) or miRNA inhibitor NC (5′CAGUACUUUUGUGUAGUACAA 3′) were purchased from GenePharma, Shanghai, China. They were used at 200 nM for transfection with Lipofectamine 2000 in cells.

### 2.5. Immunocytochemistry

Immunocytochemistry was performed as previously described [25]. Anti-M1 (A/California/04/2009 (H1N1)) monoclonal antibody (IT-003-036 M1) was purchased from Immune Technology (NYC, USA).

### 2.6. Western Blot Analysis

Western blot was performed as previously described [19]. Mouse anti-H1N1pdm M1 (A/California/04/2009 (H1N1)) monoclonal antibody (IT-003-036 M1) was purchased from Immune Technology (NYC, USA). Goat anti-PB1 (influenza A virus) polyclonal antibody (sc-17601) was purchased from Santa Cruz Biotechnology (CA, USA). Goat anti-procaspase 3 (sc-1226) and mouse anti-*β*-actin antibodies (sc-130300) were obtained from Santa Cruz Biotechnology (Santa Cruz, CA, USA). Rabbit anti-cleaved caspase 3 (#9664), rabbit anti-cleaved caspase 8 (#9496), and rabbit anti-cleaved caspase 9 antibodies (#9501) were products of Cell Signaling Technology (Boston, USA).

### 2.7. Quantitative RT-PCR

Total RNA from human bronchial epithelial cell (HBE), HEK293, HEK293T, or MDCK cells were isolated using an miRNeasy kit (Tiangen, Beijing, China), which were used for SYBR Green-Based quantitative RT-PCR (qRT-PCR; GenePharma, China) to determine copy numbers of let-7b miRNA according to the manufacturer's protocol. miRNA primers are listed as follows: let-7b: forward: 5′- TGAGGTAGTAGGTTGTGTGGTTGC-3′; reverse: 5′- TATGGTTGTTCTCGTCTCCTTCTC-3′.

RNA isolation and reverse transcription were described previously [21, 25]. Quantitative RT-PCR was done using a SYBR Green-Based quantitative RT-PCR kit (Takara, Tokyo, Japan) following manufacturer's protocol. Relative expression values were standardized by an internal glyceraldehyde-3-phosphate dehydrogenase (GAPDH) control. Fold changes of mRNA transcripts of IL-6 and the viral PB1, M gene segment, were calculated with the formula 2^(ΔC_T_  of  gene  −  ΔC_T_  of  GAPDH)^, where C_T_ is threshold cycle [25]. RT-PCR primers are listed as follows: IL-6: forward: 5′- ACTCACCTCTTCAGAACGAA -3′; reverse: 5′- CCATCTTTGGAAGGTTCAGG-3′, GAPDH: forward: 5′- ACAGTCAGCCGCATCTTCTT-3′; reverse: 5′- ACGACCAAATCCGTTGACTC-3′, M: forward: 5′- ATGGCTGGATCGAGTGAACA-3′; reverse: 5′-TTTGCTGCAATGACGAGAGG-3′, PB1: forward: 5′- TGGGAATCAACATGAGCAAA-3′; reverse: 5′- GCAGGTCCAAGGTCATTGTT-3′.

### 2.8. Luciferase Activity Assay

In total, 40 000 cells were seeded in 24-well plates 24 hrs prior to transfection. The cells were cotransfected with 0.8 *μ*g of either pmir-mu-PB1, pmir-scbl-PB1, pmir-wt-PB1, or pmir-reporter together with 40 nM of let-7b-mimic or 40 nM of miR-let-7b negative control (NC) using Lipofectamine 2000 following manufacturer's protocol. Firefly and renilla luciferase activities were measured by using dual-luciferase reporter assay (Promega, Madison,WI) 24 hrs after transfection.

### 2.9. ELISA Assay for Cytokines

Cell culture media were taken at 24 h after infection (p.i.). Cytokines were measured by commercially available enzyme-linked immunosorbent assay (ELISA) kits (Nanjing Senbeijia Biotech Company, China) following manufacturer's manual.

### 2.10. Flow Cytometric Analysis

Apoptosis was analyzed by flow cytometry using propidium iodide (PI) and the annexin-V-FITC staining kit (BD Biosciences, Vienna, Austria) following manufacturer's manual.

Data were analyzed by using FlowJo software version 7.2.5 (Tree Star Inc., Ashland, OR, USA).

### 2.11. Statistical Analysis

For statistical analysis, a two-tailed Student's *t*-test was used to evaluate the data in two groups by SPSS software (IBM SPSS, Armonk, NY). One-way ANOVA analysis was used to determine significant differences of the data in three or more groups with the 0.05 level of probability (*P* < 0.05) being considered to be statistically significant.

## 3. Results

### 3.1. Construction of Recombinant H1N1pdm Viruses with let-7b Targeting Sequence Inserted in PB1

miR-let-7b is abundant in bronchial epithelial cells. Using quantitative real-time PCR, we showed that miR-let-7b was rich in HBE cells and lowly expressed in HEK293, HEK293T, or MDCK cells (Figure 1(a)). To incorporate miR-let-7b target sites into influenza A virus, we chose the highly conserved genomic segment two of the H1N1pdm genome, which encodes PB1 of three RNA polymerase components. Without changing the polarity and the charge of amino acids, we incorporated MREs for miRNA let-7b into the open reading frame through minimal substitutions, the bases of the sequences. At one site, there were three amino acid substitutions required to generate an MRE-containing PB1 (Figure 1(b)). Primers were designed and used for amplifying cDNA of PB1 by PCR to obtain mutant PB1 cDNA, which was cloned into pDP2000, a vector plasmid for rescuing recombinant viruses by reverse genetics approach. A HEK293T and MDCK mixed culture was used for transfection with eight pDP2000 plasmids harboring cDNAs of H1N1pdm including the mutant PB1. The resultant recombinant virus was termed miRT-H1N1. A control virus, termed scbl-H1N1, was also obtained which contained the three identical amino acid substitutions in addition to a few more mutations that disrupt miR-let-7b binding to the RNA sequence but bring no more change to amino acid composition (Figure 1(b)). scbl-H1N1 will allow us to distinguish phenotypic differences due to miRNA targeting from unwanted effects potentially as a result of the three nucleotide substitutions in PB1. The influenza A genome is composed of eight vRNA segments with negative polarity. They are PB1, PB2, PA, HA, NP, NA, M, and NS genes. Viral RNA of miRT-H1N1, scbl-H1N1, or wild type H1N1pdm (wt H1N1) was purified from virus particles. We performed a two-step RT-PCR: the first step was reverse transcription, which was performed by using the Uni12 primer that is complementary to the 12 conserved nucleotides at the 3′-end of the vRNA. The reverse transcription product(s) was then amplified by PCR using segment-specific primers [24]. Thus, the products were used for sequence analysis. Sanger sequencing was performed to detect escape mutants. No sequence alterations were found in the viral RNA of miRT-H1N1, scbl-H1N1, or wild type H1N1pdm (wt H1N1) except the mutation previously designed in the MREs in PB1 gene of miRT-H1N1 and scbl-H1N1.

### 3.2. Decreased Viral Replication in Cells Infected with Recombinant Viruses with let-7b Targeting Sequences in PB1

**Figure 2:**
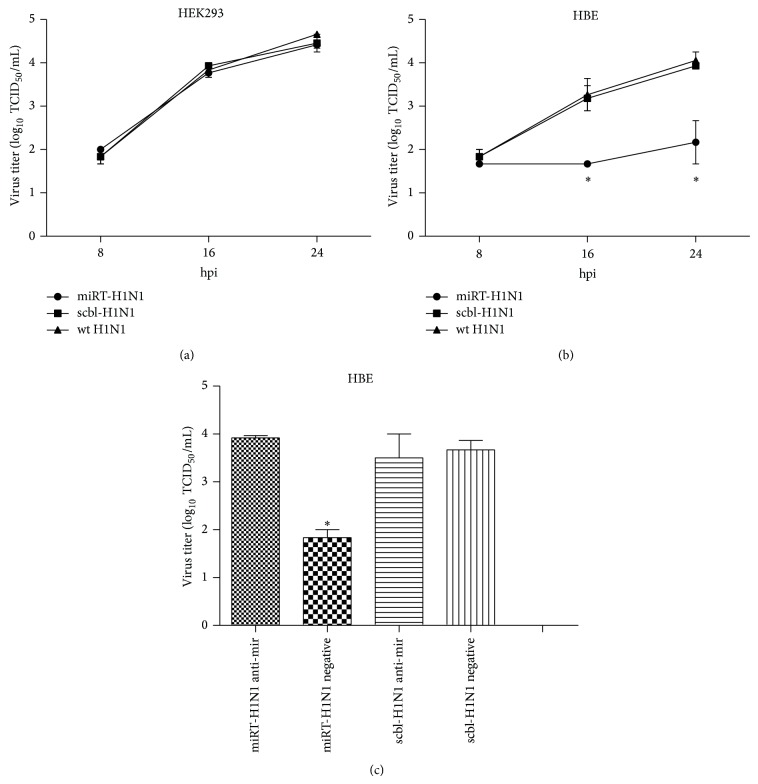
Effect of miRNA target insertions on the miRT-H1N1 replication in HEK293 and HBE cells. Replication of indicated viruses was assessed in HEK293 (a) and HBE (b) cells following inoculation at an MOI of 1. Virus titers were determined at 8, 16, and 24 h after inoculation on HEK293 and HBE cells. Mean virus titers and standard errors are shown. The asterisk indicates that replication of miRT-H1N1 was significantly different from that of scbl-H1N1 and from parental wt H1N1 in HBE cells (*P* < 0.05). (c) Inhibition of endogenous let-7b using anti-mir technology rescues miRT-H1N1 viral replication in the nonpermissive HBE cells. HBE cells were transfected with small oligonucleotides corresponding to the let-7b complementary sequence (anti-mir let-7b) or control nonspecific oligonucleotide (ctrl anti-mir). HBE cells were then infected with scbl-H1N1 or miRT-H1N1 virus. Viral titer values represent the mean ± SD of three independent experiments. Error bars: SD. The asterisk indicates that replication of miRT-H1N1 using ctrl anti-mir was significantly different from that of scbl-H1N1 and from miRT-H1N1 using anti-mir in HBE cells. ^*^ *P* < 0.05.

Human HBE or HEK293 cells were infected with miRT-H1N1, scbl-H1N1, or wild type H1N1pdm (wt H1N1) at an MOI of 1, respectively. Culture media were taken at 8, 16, and 24 hrs p.i. for infectious virus titration in HBE or HEK293 cells. As shown in Figure 2, while scbl-H1N1 and wt H1N1 replicated equally well, the replication of miRT-H1N1 was decreased significantly in infected HBE cells (Figure 2(b)). However, three viruses replicated with the infectious virus titers comparably in HEK293 cells (Figure 2(a)). Engineered H1N1pdm with an insertion of miR-let-7b targeting sequence in PB1 gene has been compromised in replication in let-7b rich HBE cells, while the mutant virus replicated similarly to parental wt virus or the mutant viruses with a defective let-7b targeting sequence in HEK293 cells. In other words, insertion of a microRNA targeting sequence could cause a cell type-dependent attenuation for a mutant H1N1pdm virus. We further examined virus replication in HBE cells, which were transfected with an inhibitor of miRNA let-7b. Anti-miR inhibitor was chemically modified, single-stranded nucleic acids that are designed to specifically bind and inhibit endogenous miRNA molecules. The HBE cells were transfected with let-7b inhibitor, prior to infection with miRT-H1N1 or scbl-H1N1. After infection, culture media were taken at 24 hrs p.i. for infectious virus titration. As shown in Figure 2(c), miRT-H1N1 replication was inhibited with a negative control inhibitor but the inhibition was reversed in the presence of anti-mir-let-7b in HBE cells. This suggests that when let-7b is suppressed by its inhibitor, miRT-H1N1 replicated comparably to the scbl-H1N1 in HBE cells.

### 3.3. Decreased Viral Proteins and RNA in miRT-H1N1-Infected HBE Cells

**Figure 3:**
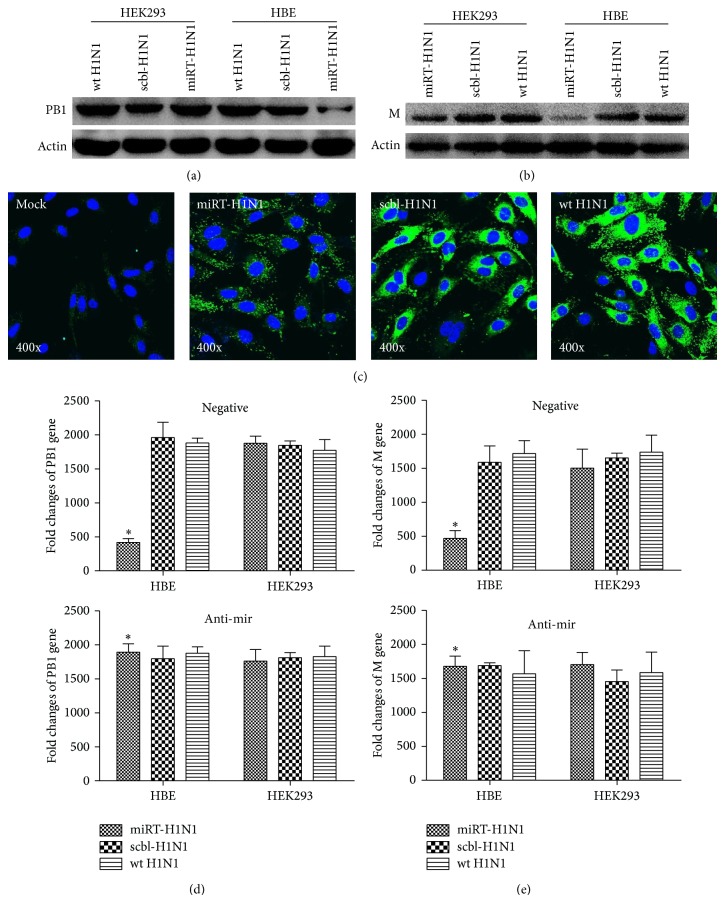
let-7b expression in cells inhibited viral protein and gene expression of miRT-H1N1. Endogenous let-7b in HBE cells diminished viral PB1 gene (a) and M gene (b) expression with miRT-H1N1 infection. HBE or HEK293 cells were infected with miRT-H1N1, scbl-H1N1, or unmodified wt H1N1 at MOI 1 and, at 24 h p.i., cell lysates were examined by western blot analysis with antibodies against H1N1 PB1, M, or actin. The expression of PB1 or M is relative to that of actin. (c) IFA of H1N1 M in HBE cells infected with miRT-H1N1, scbl-H1N1, or wt H1N1 at 24 h p.i. after infection. Reduced expression of M from miRT-H1N1 was visualized by IFA in HBE cells using a H1N1 M-specific monoclonal antibody, and representative photomicrographs are shown at 400x magnification. HBE or HEK293 cells were transfected with anti-mir let-7b or ctrl anti-mir and the amount of viral PB1 (d) and M (e) was measured with quantitative PCR from infected HBE and HEK293 cells at 24 h after infection. Human GAPDH was an internal control. Data are mean ± SD of 3 independent experiments. ^*^ *P* < 0.05.

HBE or HEK293 cells were infected with miRT-H1N1, scbl-H1N1, or wild type H1N1pdm (wt H1N1) at an MOI of 1, respectively. Cell lysates were prepared at 24 hrs p.i. for SDS-PAGE and proteins were analyzed with western blot analyses using antibodies specific for viral proteins. Expression of PB1 and M protein of H1N1pdm was significantly reduced in miRT-H1N1-infected HBE cells but not in scbl-H1N1-infected cells in comparison with wt H1N1 infection (Figures 3(a) and 3(b)). However, the levels of PB1 and M were comparable in HEK293 cells infected with either miRT-H1N1 or scbl-H1N1 and wt H1N1 in HEK293 cells (Figures 3(a) and 3(b)). HBE cells, which were mock-infected or infected with miRT-H1N1 or scbl-H1N1 and wt H1N1, were fixed with paraformaldehyde at 24 hrs p.i. and stained with anti-viral M antibody in an IFA. As shown in Figure 3(c), the level of M protein was significantly lower in miRT-H1N1-infected cells than in scbl-H1N1 or wt H1N1-infected cells.

Total RNA was extracted from HBE and HEK293 cells infected with miRT-H1N1, scbl-H1N1, or wt H1N1 to measure fold changes of viral genes by quantitative PCR at 24 hrs p.i. In HBE cells infected with miRT-H1N1, the fold changes of viral PB1 (Figure 3(d)) and M (Figure 3(e)) genes were significantly lower in miRT-H1N1-infected HBE cells than those in scbl-H1N1 or wt H1N1-infected cells. On the other hand, the fold changes of either PB1 (Figure 3(d)) and M (Figure 3(e)) genes were comparable in HEK293 cells infected with either miRT-H1N1 or scbl-H1N1 and wt H1N1-infected cells. The HBE and HEK293 cells were transfected with let-7b inhibitor, prior to infection with miRT-H1N1, scbl-H1N1, or wt H1N1. After infection, culture media were taken at 24 hrs p.i. to measure fold changes of viral genes by quantitative PCR. As shown in Figures 3(d) and 3(e), the fold changes of either PB1 (Figure 3(d)) or M (Figure 3(e)) genes were reversed in the presence of anti-mir-let-7b in HBE cells.

### 3.4. Host Apoptotic and Cytokine Responses

**Figure 4:**
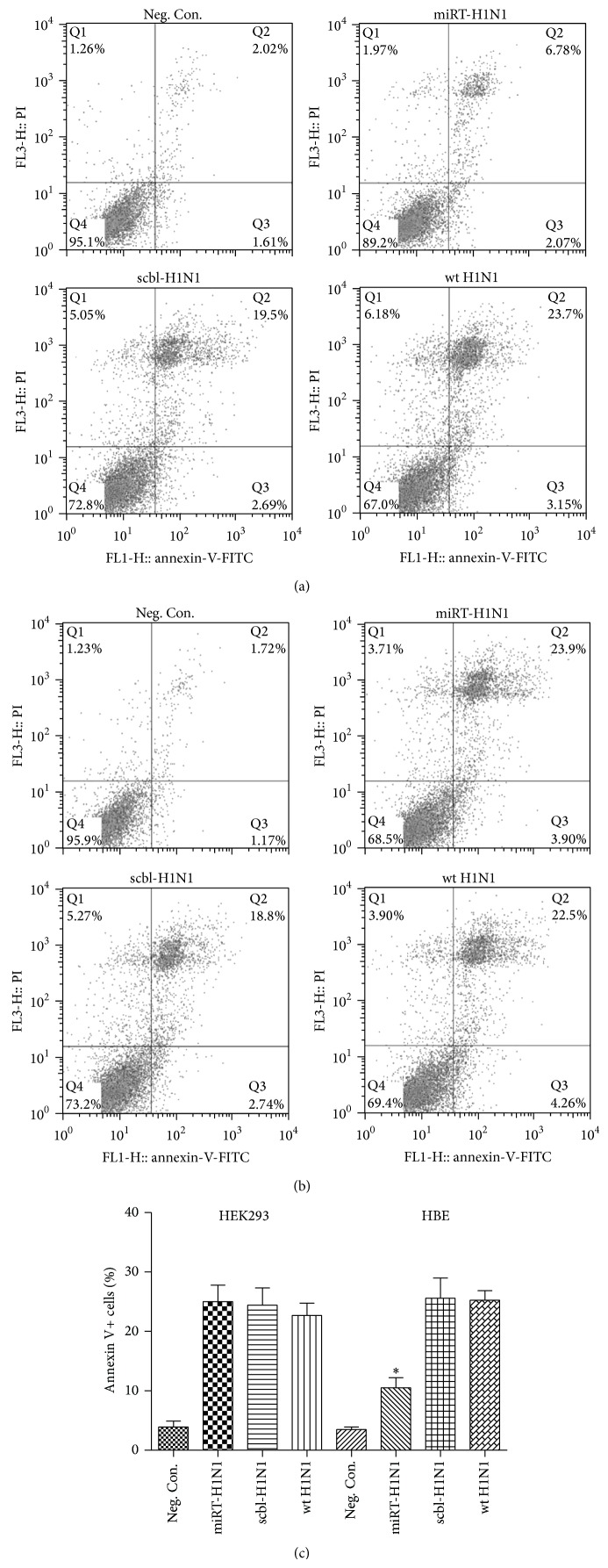
Detection of apoptosis cells with flow cytometry. Cells were prepared from miRT-H1N1, scbl-H1N1, or wt H1N1-infected and uninfected HBE (a) and HEK293 (b) cells at 24 h after infection. The percentage of total apoptosis cells (late apoptotic/necrotic cells and early apoptotic cells) was shown after analysis by flow cytometry. Data were expressed as mean ± SD. ^*^ *P* < 0.05. Apoptosis of cells in different conditions was shown in four-quadrant diagram. The lower left quadrant contains the normal (double negative) population, the upper left quadrant contains the dead (annexin V−/PI+) population, the upper right quadrant contains the late apoptotic/necrotic (annexin V+/PI+) cells, and the lower right quadrant contains the early apoptotic (annexin V+/PI−) cells.

We examined cell death caused by mutant viruses inserted with the miR-let-7b targeting sequence. MDCK cells were mock-infected, infected with miRT-H1N1, scbl-H1N1, or wt H1N1, and digested with trypsin at the indicated time point for flow cytometry with FITC-conjugated annexin V and PI. In HBE cells, miRT-H1N1 infection induced apoptosis and annexin V+ cells were 8.85%, significantly lower than the apoptotic cells in infection by wt H1N1 (26.85%) and scbl-H1N1 (22.19%) (Figure 4(a)). On the other hand, the apoptotic cells were comparable in HEK293 cells infected with either miRT-H1N1 or scbl-H1N1 and wt H1N1. (Figure 4(b)).

**Figure 5:**
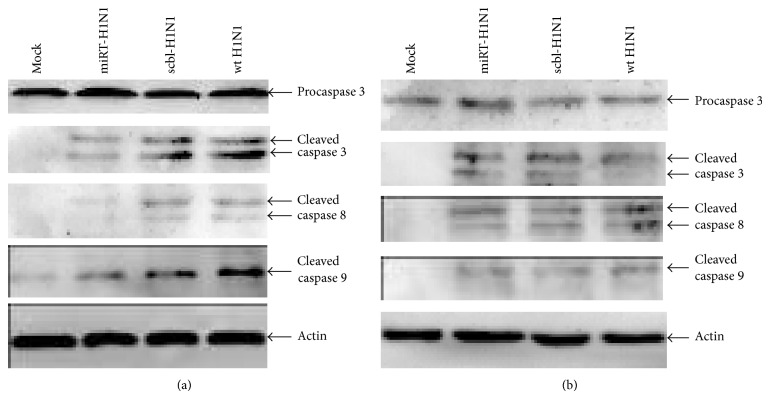
Activation of apoptotic proteins in H1N1-infected HEK293 and HBE cells. Cell lysates were prepared from miRT-H1N1, scbl-H1N1, or wt H1N1-infected and uninfected HBE (a) and HEK293 (b) cells at 24 h after infection. The cell lysates were resolved with 10–15% SDS-PAGE. Proteins were transferred to PVDF membranes for western blot analyses. Analyses were performed at least three times for each protein. Cleavage of caspases 3, 8, and 9 was shown.

We also examined caspase activation in infection using cell lysates prepared from infected cells and analyzed by western blot with antibodies specific for cleaved caspases 8, 9, and 3. The results showed that apoptosis occurred as we observed cleaved/activated caspases 8 and 9 as well as the emergence of downstream executioner caspase 3, which eventually destroyed the infected HBE or HEK293 cells. Expression of cleaved caspases 8, 9, and 3 protein of H1N1pdm was significantly reduced in miRT-H1N1-infected HBE cells, but not in scbl-H1N1-infected cells in comparison with wt H1N1 infection (Figure 5(a)). However, the levels of cleaved caspases 8, 9, and 3 protein were comparable in HEK293 cells infected with either miRT-H1N1 or scbl-H1N1 and wt H1N1 in HEK293 cells (Figure 5(b)).

**Figure 6:**
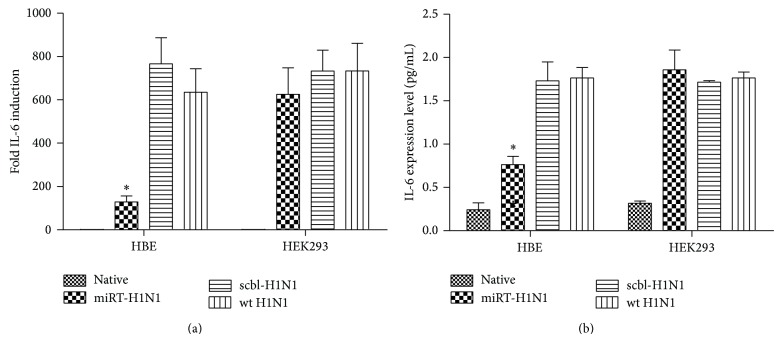
Induction of IL-6 in response to let-7b targeted H1N1. (a) HEK293 and HBE cells infected with miRT-H1N1, scbl-H1N1, or wt H1N1 viruses at an MOI of 1 and analyzed for IL-6 mRNA at 24 hpi. (b) Protein level of interleukin- (IL-) 6 was evaluated by enzyme-linked immunosorbent assay (ELISA). Data from (a) and (b) are representative of three independent experiments with three to four samples per group. ^*^ *P* < 0.05.

Proinflammatory cytokine IL-6 has been reported to be dominant in H1N1pdm infected patients or HBE cells and may be involved in viral pathogenesis in infection. To evaluate how cytokine responses were affected in infection with the mutant viruses, total RNA was prepared for real-time RT-PCR with IL-6 primers. In HBE cells infected with scbl-H1N1 or wt H1N1, IL-6 was upregulated to over 600- and 700-fold, respectively, while, in infection with miRT-H1N1, IL-6 induction was significantly lower (Figures 6(a) and 6(b)). Culture media were taken for measuring IL-6 concentrations after infection. Significantly lower IL-6 was detected in infection with miRT-H1N1 compared with scbl-H1N1 or wt H1N1 (Figures 6(a) and 6(b)). On the other hand, in HEK293 cells, IL-6 was upregulated at comparable levels both in mRNA transcripts (Figures 6(a) and 6(b)) and concentrations (Figures 6(a) and 6(b)) in infections with miRT-H1N1, scbl-H1N1, or wt H1N1, indicating that proinflammatory cytokine IL-6 was induced distinctly with the mutant virus only in HBE cells.

### 3.5. miR-let-7b Targeted PB1 Containing miR-let-7b–MRE

**Figure 7:**
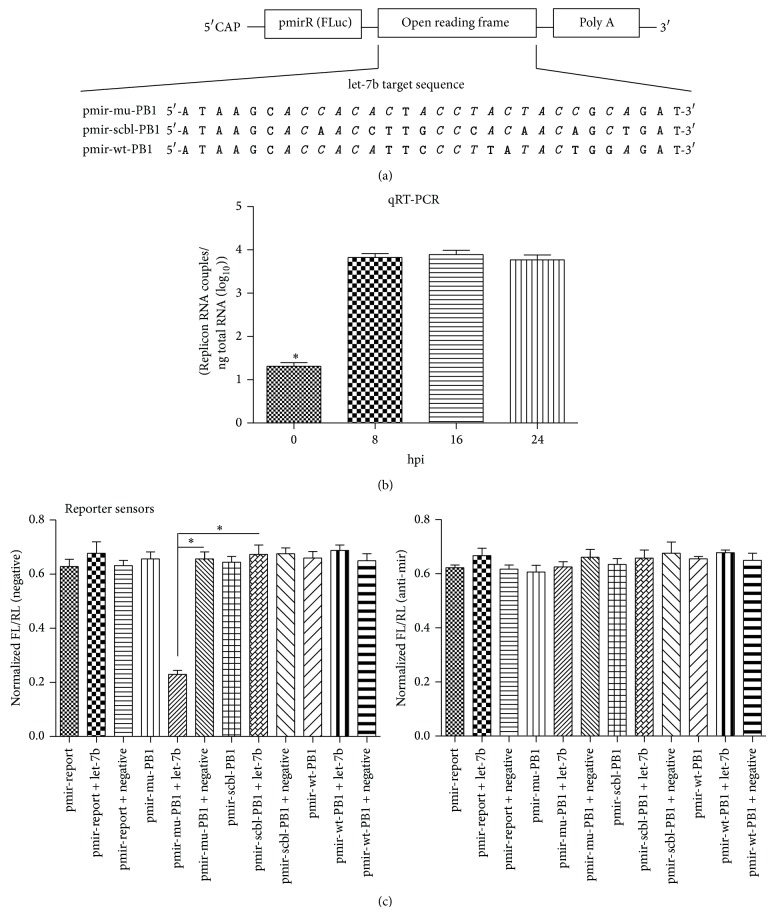
Ectopic expression of let-7b inhibits the luciferase activity of reporter. (a) Schematic diagram of various sensors carrying 1 copy of MRE in the open reading frame of mRNAs. MREs that are complementary to let-7b are in italic, and mismatched parts are in bold. (b) Representative quantitative RT-PCR analysis of let-7b miRNA expression levels observed in let-7b-mimic-transfected HEK293 cells. (c) HEK293 cells were transfected with anti-mir let-7b or ctrl anti-mir. For reporter sensors, firefly luciferase (FLuc) was normalized to internal control renilla Luc (RLuc) for each sensor. The ratio of FLuc to RLuc in each group was determined. Data are mean ± SD from experiments repeated 3 times in triplicate. Error bars denote means ± SD from triplicate experiments; similar results were obtained in three independent experiments. ^*^ *P* < 0.05.

To evaluate the interaction of miR-let-7b and its MRE, we used pmir-reporter as backbone to generate a reporter plasmid carrying firefly luciferase (FLuc) and miR-let-7b MREs, which was termed pmir-mu-PB1 (Figure 7(a)). miR-let-7b was at low levels in HEK293 cells (Figure 1(a)). When HEK293 cells were transfected with an let-7b-mimic, overexpression of let-7b could be detected (Figure 7(b)). Cells were cotransfected with pmir-reporter, pmir-mu-PB1, or pmir-scbl-PB1, respectively, together with either let-7b-mimic or let-7b negative control. 12 hrs after transfection, lysates were prepared from the cells for luciferase assays. As shown in Figure 7(c), expression of miR-let-7b, but not miR-let-7b control, inhibited pmir-mu-PB1 by more than 50%. On the other hand, miR-let-7b had no inhibitory effect on the pmir-reporter, pmir-wt-PB1, or pmir-scbl-PB1 (Figure 7(c)), which suggests that miR-let-7b targets specifically the PB1 gene with let-7b targeting sequences inserted. The HEK293 cells were transfected with let-7b inhibitor, prior to cotransfection with pmir-reporter, pmir-mu-PB1, or pmir-scbl-PB1, respectively, together with either let-7b-mimic or let-7b negative control. 12 hrs after transfection, lysates were prepared from the cells for luciferase assays. As shown in Figure 7(c), the inhibited pmir-mu-PB1 by miR-let-7b was reversed in the presence of anti-mir-let-7b in HEK293 cells.

While the inclusion of target elements for miR-let-7b targeting the mRNA of the PB1 gene of H1N1 did significantly attenuate the virus in the HBE cells and increase the therapeutic index of H1H1, mutation of these target elements is always of potential concern. Because only ~24 nt of sequence insert provided the entire basis of the reduced toxicity for this virus, we looked at the potential of this virus to mutate the sequence insert. This virus was adapted to HEK293 cell lines by continuous passage for about 10 passages and the miRT insert and flanking region were cloned into PGM-T vector and sequenced, but no sequence alterations in the miRT insert were found, suggesting that the flexible nature of miRNA targeting combined with the opposing rigid conservation of the open reading frame may prevent reversion and add to the safety.

## 4. Discussion

Each cell type displays a particular miRNA repertoire. Viral replication could be controlled by these cellular miRNAs with different pathogenic profiles in a cell-specific manner [26, 27].

In the influenza A virus, the acidic subunit PA and the two basic subunits PB1 and PB2 are the three largest vRNAs which encode the three subunits of the RNA-dependent RNA polymerase [28]. We chose the highly conserved segment two of H1N1, which encodes PB1 of the three main polymerase components. Here, we show that the pathogenicity of H1N1 virus can be selectively attenuated by inserting target elements for microRNA let-7b in the viral genome in HBE which is rich in miRNA (let-7b). By using a well validated let-7b-based cell line model system, we demonstrated the capacity of cell type specific microRNAs to control H1N1 replication in vitro. These studies also confirmed that the mechanism of H1N1 attenuation is to incorporate MREs into the viral open reading frame of PB1 which is involved in the decrease of RNA replication. Our results showed that detargeting by let-7b is effective as demonstrated by strongly reduced virulence. Virus titers in HEK293 cells showed similar replication kinetics for miRT-H1N1 compared to the unattenuated control virus (wt H1N1) or scbl-H1N1, indicating that the reduced virulence observed after miRT-H1N1 infection is due to let-7b induced attenuation of the virus.

Apoptosis is primarily a host defense mechanism limiting virus replication [29, 30]. Our study reports that the number of apoptotic cells and the activation of the initiator and executioner caspases by miRT-H1N1 infection are significantly downregulated compared to wt H1N1 and scbl-H1N1 cells in HBE cells. The characterization of the MRE-containing influenza viral strains confirmed changes in viral tropism and a normal cellular response to infection, demonstrating robust cytokine. IL-6 was detected in the HEK293 cells following infection with miRT-H1N1, scbl-H1N1, or wt H1N1 at 24 hpi. Little IL-6 was detected in miRT-H1N1-infected HBE samples. These results are in line with the previous finding that the expression of cytokines correlates with the rate of H1N1 replication [31].

The perfect complementarity between the target sequence and cellular miRNA is a critical factor for miRNA target recognition [32, 33]. Deletions or mutations within the region of the miRNA target sequence can contribute escape of virus from the miRNA-mediated suppression [34, 35]. The flexible nature of miRNA targeting combined with the opposing rigid conservation of the open reading frame may prevent reversion and add to the safety [15]. We demonstrate that selective pressure does not provide any change in the sequence of miRT-H1N1 in vitro.

In conclusion, by the inclusion of a single copy of the target for miRNAs expressed in the HBE, we demonstrate that the virulent phenotype of the influenza virus is decreased or almost completely abolished in vitro. The miRNA targeting approach can control influenza virus tropism. This finding develops live attenuated virus vaccines against various influenza viruses. The potential benefit of incorporated MREs into the viral open reading frame remains to be investigated for the perspective of the safety and stability of new vaccine candidates carrying miRNA targets. To further examine the protection afforded by miRT-H1N1 immunizations, we will perform studies on an animal model.
